# Study of Chitosan-Stabilized Ti_3_C_2_T*_x_* MXene for Ultrasensitive and Interference-Free Detection of Gaseous H_2_O_2_

**DOI:** 10.1021/acsami.3c05314

**Published:** 2023-06-23

**Authors:** Jelena Isailović, Ana Oberlintner, Uroš Novak, Matjaž Finšgar, Filipa M. Oliveira, Jan Paštika, Zdeněk Sofer, Nikola Tasić, Rui Gusmão, Samo B. Hočevar

**Affiliations:** † Department of Analytical Chemistry, National Institute of Chemistry, Hajdrihova 19, 1000 Ljubljana, Slovenia; ‡ Department of Inorganic Chemistry, University of Chemistry and Technology Prague, Technická 5, 16628 Prague, Czech Republic; § International Postgraduate School Jožef Štefan, Jamova cesta 39, 1000 Ljubljana, Slovenia; ∥ Department of Catalysis and Chemical Reaction Engineering, National Institute of Chemistry, Hajdrihova 19, 1000 Ljubljana, Slovenia; ⊥ Faculty of Chemistry and Chemical Engineering, University of Maribor, Smetanova ulica 17, 2000 Maribor, Slovenia

**Keywords:** Ti_3_C_2_T*_x_* MXene, chitosan, hydrogen peroxide, gas sensor, cyclic voltammetry

## Abstract

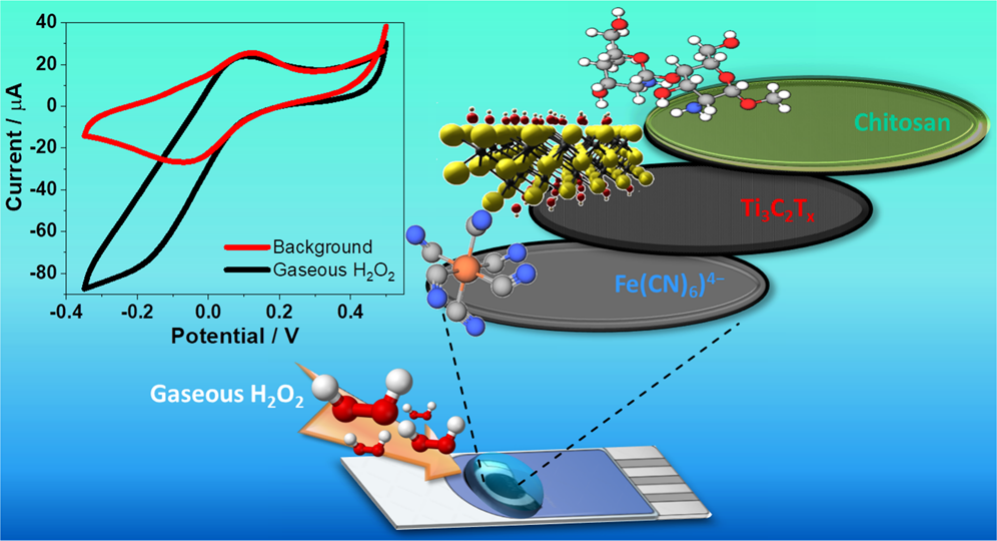

The development of sensitive, selective, and reliable gaseous hydrogen peroxide (H_2_O_2_) sensors operating at room temperature still represents a remaining challenge. In this work, we have investigated and combined the advantageous properties of a two-dimensional Ti_3_C_2_T*_x_* MXene material that exhibits a large specific surface area and high surface activity, with favorable conducting and stabilizing properties of chitosan. The MXene–chitosan membrane was deposited on the ferrocyanide-modified screen-printed working carbon electrode, followed by applying poly(acrylic acid) as an electrolyte and accumulation medium for gaseous H_2_O_2_. The sensor showed highly sensitive and selective electroanalytical performance for detecting trace concentrations of gaseous H_2_O_2_ with a very low detection limit of 4 μg m^–3^ (4 ppbv), linear response in the studied concentration range of 0.5–30.0 mg m^–3^, and good reproducibility with an RSD of 1.3%. The applicability of the sensor was demonstrated by point-of-interest detection of gaseous H_2_O_2_ during the real hair bleaching process with a 9 and 12% H_2_O_2_ solution.

## Introduction

In recent years, MXenes have attracted immense attention due to their high metallic conductivity, hydrophilicity, low diffusion barrier, high ion transport properties, biocompatibility, large surface area, and ease of functionalization and thus can serve as a remarkable interface for the development of next-generation sensing strategies.^1−4^ This novel class of two-dimensional (2D)-layered materials consists of transition metal carbides or nitrides, with the general formula M_*n*+1_X*_n_*T*_x_*, where M stands for the transition metal (e.g., Ti, Zr, V, Cr, Nb), X is carbon or nitrogen, and T corresponds to different terminal groups, e.g., O, F, OH, and Cl.^3^ Etching of A elements in the MAX phase (A is a p-block element) yields the designated MXenes, as introduced by Gogotsi et al.^5^ To date, more than twenty different MXene compounds have been successfully synthesized from the precursor MAX phases by etching and exfoliation methods,^3,6,7^ resulting in surface-functionalized MXenes with abundant terminal groups that account for their hydrophilic nature. As such, MXenes can selectively absorb biomolecules and gas molecules through morphology control and surface modification.^8−10^ The drawback is that MXenes are highly susceptible to oxidative degradation, leading to impaired functionality and inefficacy. Research efforts related to the specifics of synthesis protocols, storage conditions, or storage media appear to offer some comfort regarding their stability during preparative steps.^11,12^ However, for electroanalytical applications, when MXenes are incorporated on the supporting electrodes and subjected to measurements in wide potential windows, an additional application obstacle arises from their innate and irreversible electro-oxidation.^13^ The existing literature does not yet offer a convincing solution to this problem, which was also addressed in this study.

H_2_O_2_ is an important molecule that plays a vital role in various fields, such as medicine, clinical diagnostics, biotechnology, chemical synthesis, textile, wood, food, and pharmaceutical industries in the production of cosmetics, dyes, detergents, etc.^14−17^ Besides its main property as an oxidant, H_2_O_2_ is a key molecule in numerous biological systems involving the enzymatic activity of, e.g., oxidases and peroxidases.^18^ It is essential as a signaling molecule in the biological processes of the immune system, apoptosis, and root growth.^19,20^ As a biomarker, H_2_O_2_ is associated with oxidative stress^21,22^ and, as a gaseous molecule, with several diseases such as lung cancer and other pulmonary disorders.^23−27^ In atmospheric processes, gaseous H_2_O_2_ accompanies reactions involving UV radiation, free radicals, and the transformation of gaseous pollutants. Its occurrence may also indicate the presence of relatively labile peroxo-explosives.^28^ Conventional analytical methods typically used for the determination of H_2_O_2_ include spectroscopy,^29−31^ chemiluminescence,^24,32−34^ colorimetry,^35^ fluorimetry,^36^ gas chromatography–mass spectrometry (GC–MS),^37^ and others.^38,39^ Most of these techniques can achieve satisfactory detection limits, sensitivity, and selectivity; however, expensive and robust equipment, limited portability, and relatively complex sample preparation are persistent challenges that must be overcome. On the other hand, electrochemical sensing offers an attractive alternative to conventional methods, especially in terms of low-cost and compact instrumentation, portability, capability for point-of-interest detection, sensitivity, and relative ease of operation.^40^ Many powerful electrochemical sensors have been developed for the detection of H_2_O_2_ in liquid samples, incorporating numerous redox modifiers, metal, metal oxide, and nonmetallic nanoparticles, enzymes, polymeric membranes, and combinations thereof.^41,42^ However, to the best of our knowledge, there are very few reports of electrochemical sensors for detecting considerably more challenging gaseous H_2_O_2_.^43−48^

In this work, we have investigated the integration of Ti_3_C_2_T*_x_* MXene into the sensing membrane to construct a disposable, ultrasensitive, and interference-free electrochemical sensor for onsite detection of gaseous H_2_O_2_. Moreover, through a synergistic effect between Ti_3_C_2_T*_x_* MXene and chitosan membrane, we successfully stabilized the 2D-layered MXene. We exploited the electrocatalytic characteristics of MXene by combining it with a ferrocyanide-modified working carbon electrode and poly(acrylic acid), the latter simultaneously serving as a suitable accumulation and electrolyte medium for gaseous H_2_O_2_. We characterized the sensing interface, including the interaction between chitosan and MXene and the stabilization of MXene.

## Experimental Section

### Apparatus

Electrochemical studies were carried out using a portable potentiostat/galvanostat PalmSens4 (PalmSens BV, Houten, Netherlands) in combination with a cable connector for screen-printed electrodes (DRP-CAC 71606, Metrohm DropSens, Herisau, Switzerland) and PSTrace 5.9 software (PalmSens BV). The supporting screen-printed electrode system consisted of a ferrocyanide-bulk-modified carbon working electrode (FCN-SPCE, the diameter of the working electrode was 4 mm), a silver quasi-reference electrode, and a carbon counter electrode (DRP-F10, Metrohm DropSens) designed to work with microvolume solution droplets.

### Reagents and Solutions

Methanol (VWR International, Radnor, Pennsylvania), ethanol, benzene (Carlo Erba Reagents, Milano, Italy), H_2_O_2_ (30%), ammonia, formaldehyde, nitric acid (65%), glycerol, sulfuric acid (95–98%) (all from Merck, Rahway, New Jersey), phenol, high-molecular-weight chitosan (310–375 kDa; ≥75% deacetylated), lactic acid (85%), and sodium nitrite (97%) (all from Sigma-Aldrich, St. Louis, Missouri) were of the analytical grade purity. Pure O_2_ (N5.0) was purchased from Messer (Frankfurt, Germany). The chitosan blend (CHI) was prepared as follows: 1.5% (w/v) chitosan and 30.0% (w/w) glycerol were dissolved in 100.0 mL of 1.0% (v/v) aqueous solution of lactic acid, and the mixture was continuously stirred (Ika, Staufen, Germany) overnight at room temperature (24 °C), as reported previously.^49^ Test solutions yielding the desired gaseous-phase H_2_O_2_ concentrations above the solution were prepared in 100.0 mL glass flasks according to Henryʼs law^50^ in the presence of the ambient atmosphere, i.e., no purging was applied. The corresponding Henry constants were given in ref (50). All solutions used in this work were prepared using water purified with the Elix 10/Milli-Q Gradient unit (Millipore, Bedford).

### Synthesis and Characterization of the Ti_3_C_2_T*_x_* MXene

For MXene synthesis, 5.0 g of Ti_3_AlC_2_ MAX phase material (Jinzhou Haixin Metal Materials, China) was immersed in 250.0 mL of hydrofluoric acid solution (Sigma-Aldrich) with 40.0 vol % and stirred continuously for four days. Then, the sample was stirred for two days in lithium fluoride/hydrochloric acid. Finally, the sample was subjected to iterative cycles of centrifugation and redispersement in water until obtaining a neutral pH value in the residual water. The obtained sample was dried for 24 h in a vacuum oven set at 50 °C.

The morphology of Ti_3_C_2_T*_x_* MXene was examined by scanning electron microscopy (SEM) with a field emission gun (FEG) electron source (Tescan Lyra dual-beam microscope) at 5 kV acceleration voltage. Elemental composition was investigated by energy-dispersive X-ray spectroscopy (EDXS) using an X-Max^N^ detector from Oxford Instruments at 20 kV acceleration voltage. For both SEM and EDXS analyses, the colloidal suspension of Ti_3_C_2_T*_x_* MXene was drop-cast on carbon tape. STEM was performed with the same instrument as SEM but with a STEM sample holder. Diluted suspension of Mxene was drop-cast on a 200 mesh Cu TEM grid and then dried. STEM measurements were carried out using a 30 kV electron beam.

X-ray diffraction (XRD) measurements were carried out using a Bruker D8 Discoverer (Bruker, Germany) diffractometer in Bragg–Brentano parfocal geometry equipped with a Cu Kα radiation source (λ = 0.15418 nm, *U* = 40 kV, and *I* = 40 mA). The diffraction pattern was acquired in the scan range of 2θ from 5 to 70° at room temperature. The data were evaluated by HighScore Plus 4.9 software. For the sample preparation, 3.0 μL of a colloidal suspension of Ti_3_C_2_T*_x_* MXene was drop-cast on a Si/SiO_2_ substrate, followed by evaporation of the solvent at room temperature.

An InVia Raman microscope (Renishaw, England) was used for Raman spectroscopy measurements in backscattering geometry with a CCD detector. An Nd:YAG laser (532 nm, 50 mW) with a 2400 line mm^–1^ diffraction grating, an applied power of 1.25%, and a 20x objective were used for the measurements. The Raman spectrum was collected with 200 accumulations in the Raman range of 100–800 cm^–1^ at room temperature. For the sample preparation, the same procedure was used as in the case of XRD measurements.

Atomic force microscopy (AFM) measurements were carried out on a Ntegra Spectra from NT-MDT in a tapping mode using a cantilever with a strain constant of 1.5 kN m^–1^ equipped with a standard silicon tip with a curvature radius less than 10 nm. Sample suspensions of exfoliated material were prepared and drop-cast on a freshly cleaved mica substrate. The measurements were carried out under ambient conditions with a scan rate of 1 Hz and 512 scan lines.

### Modification of the Working Electrode and Fabrication of the Sensor

The working electrode was first modified with Ti_3_C_2_T*_x_* MXene. Before the modification, the MXene solution was sonicated and stirred for 3 min, followed by drop-casting 1.0 μL of the 250.0 mg mL^–1^ solution on the surface of the FCN-SPCE. After drying in air for 10 min, an 8.0 μL droplet of chitosan blend (CHI, from the Reagents and Solutions section) was applied to the working electrode, i.e., on the top of the MXene layer. After drying under laboratory conditions overnight, the whole screen-printed electrode system was modified via drop-casting 20.0 μL of a viscous mixture consisting of 1.0 g of polyacrylic acid (450.000 g mol^–1^; Sigma-Aldrich) and 10.0 mL of 0.1 M phosphate buffer solution (pH = 7.2), resulting in a polyacrylic gel-like accumulation/electrolyte membrane.

### XPS Measurements

XPS measurements were performed with a Supra plus device (Kratos, Manchester, UK) equipped with an Al Kα excitation source. During the spectra acquisition, the charge neutralizer was on. The binding energy scale was corrected using the C–C/C–H peak at 284.8 eV in the C 1s spectra. The screen-printed electrodes were attached to the sample holder using carbon tape. Measurements were carried out at a 90° take-off angle at a pass energy of 20.0 eV and a step of 0.1 eV. The analysis area was 300 by 700 μm. Spectra were measured and processed using ESCApe 1.5 software (Kratos).

### ToF-SIMS Measurements

ToF-SIMS measurements were performed with an M6 device (IONTOF, Münster, Germany). During the spectra acquisition, the flood gun was on. The spectra were calibrated using the peak for CH_3_^+^ at *m*/*z* 15.02, C_2_H_3_^+^ at *m*/*z* 27.02, and C_4_H_7_^+^ at *m*/*z* 55.05. The 30.0 keV Bi^+^ with a target current of 1.2 pA was employed as the primary ion beam. Sputtering was performed on 500 by 500 μm using the 5.0 keV Ar_1200_^+^ gas cluster ion beam (GCIB), while the analysis was carried out on a 300 by 300 μm in the center of the sputter crater. Spectra were measured and processed, and 3D profiles were constructed using SurfaceLab 7.3 software (IONTOF). The depth (z-scale) of the sputter crater created by the ToF-SIMS sputtering was analyzed using a DektakXT stylus profilometer (Bruker, Karlsruhe, Germany).

### Electrochemical Measurements

Cyclic voltammetric measurements were carried out in a potential range of +0.50 to −0.35 V (unless otherwise specified) using a scan rate of 100 mV s^–1^ vs Ag quasi-reference electrode. The gas sensor attached to the electrode holder was inserted tightly into the narrow opening of the chamber, created specifically for the dimensions of a screen-printed electrode, in the area above the solution. All measurements were performed at room temperature (22–23 °C) in a model atmosphere (unless otherwise specified) above the corresponding H_2_O_2_ solution in the presence of atmospheric gases. For studying the stabilization effect of chitosan on Ti_3_C_2_T*_x_* MXene, square-wave voltammetry was used in the range of −0.5 to +1.0 V vs Ag quasi-reference electrode.

## Results and Discussion

### Characterization of Ti_3_C_2_T*_x_* MXene

The Ti_3_C_2_T_*x*_ MXene was obtained by selectively etching the Ti_3_AlC_2_ MAX phase as described in the Experimental Section and illustrated in Scheme S1. The physical and chemical properties of the obtained Ti_3_C_2_T*_x_* MXene were investigated by X-ray diffraction (XRD), Raman spectroscopy, scanning electron microscopy (SEM) combined with X-ray spectroscopy (EDXS), and atomic force microscopy (AFM).

**Figure 1:**
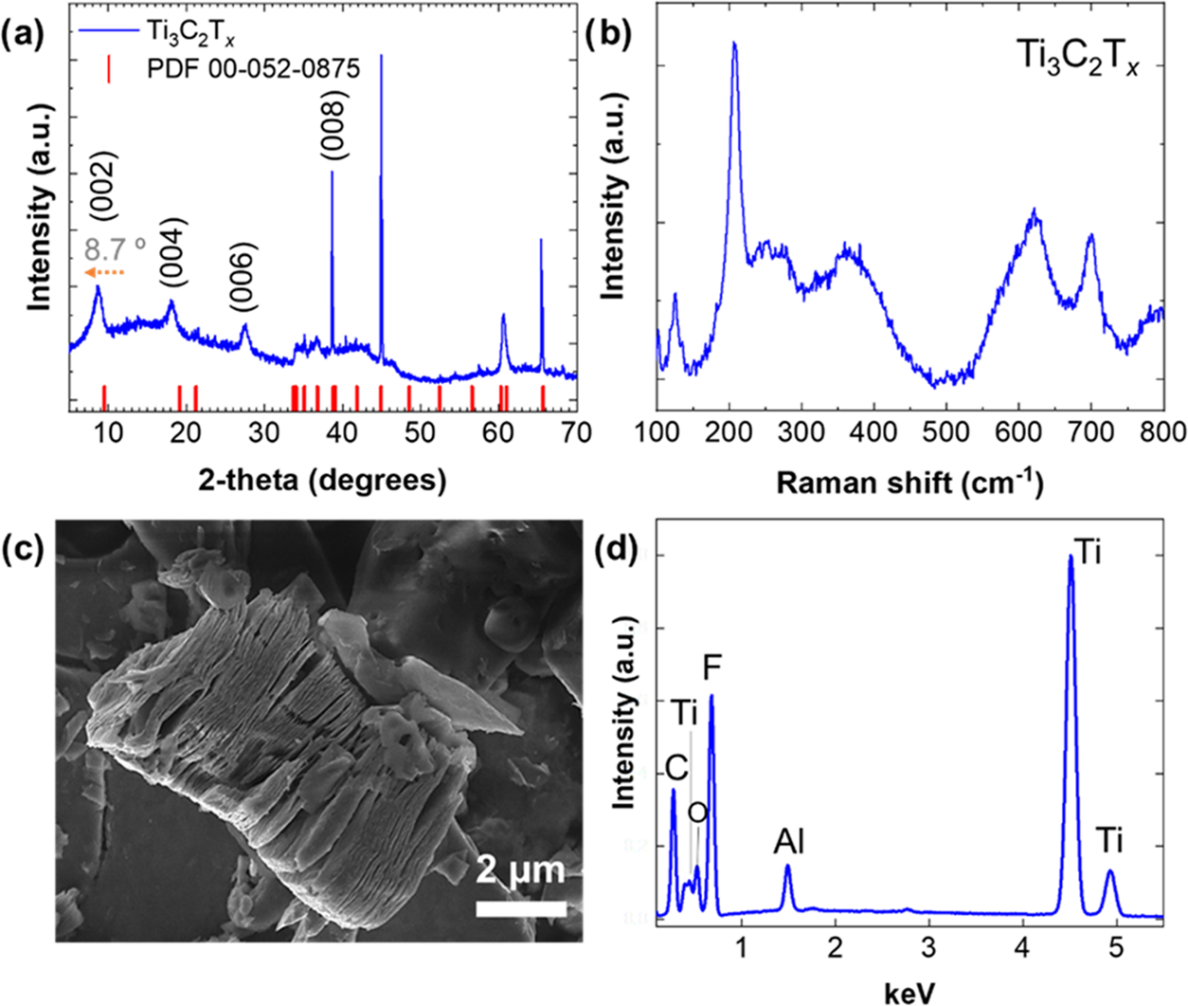
Structural and morphological characterization of Ti_3_C_2_T*_x_* MXene by XRD (a), Raman spectroscopy (b), SEM (c), and EDXS (d).

The XRD pattern of Ti_3_C_2_T_*x*_ MXene is shown in Figure 1a, and its successful synthesis is confirmed by the presence of characteristic diffraction peaks at 8.7, 18.2, 27.6, and 38.6°, which are related to the (002), (004), (006), and (008) planes of this MXene, respectively.^51^ For a better understanding of the successful synthesis of Ti_3_C_2_T_*x*_, the powder diffraction file (PDF) of its precursor MAX phase Ti_3_AlC_2_ (PDF 00-052-0875) was included for comparison. The selective etching of aluminum (Al) is confirmed by a noticeable shift in the (002) diffraction of Ti_3_AlC_2_ from 9.5 to 8.7° in Ti_3_C_2_T_*x*_. This downshift not only signifies the removal of Al but also indicates the introduction of surface terminations (T_*x*_ = −F, −O) in the structural composition of Ti_3_C_2_T*_x_*.

The Raman spectrum in Figure 1b is in agreement with previous studies^52,53^ on the vibration modes of Ti_3_C_2_T_*x*_ flakes because typical vibrations related to titanium *A*_1*g*_(Ti, C, O) at 206.5 cm^–1^ and broad peaks in the region of 230–470 cm^–1^ assigned to in-plane (*E*_*g*_) modes of surface groups attached to Ti atoms are identified. In the 580–730 cm^–1^ region, also designated as the carbon region, the peaks occurring at 621.3 and 700.3 cm^–1^ are associated, respectively, with the vibrations of Ti (*E*_*g*_) and C (*A*_1*g*_). The peak at 126.1 cm^–1^ is a characteristic resonant peak of laser use during the measurements.^53^ The numerical data regarding the identified vibrational modes are summarized in Table S1 and are compared with the reported literature.^52,53^ The observed deviations between the Raman spectrum obtained in this work and those reported in the literature (see Table S1) can be attributed to differences arising from material purities and the presence and abundance of terminal groups. Additionally, deviations between experimental results from stacked sheets and theoretical calculations performed on residual-stress-free monosheet models should also be taken into consideration.^52^ SEM analysis showed that most Al layers of the Ti_3_AlC_2_ MAX phase were eliminated after the etching procedure, obtaining the typical accordion-like morphology of the multilayer Ti_3_C_2_T*_x_* MXene (Figure 1c).^51^ The EDXS spectra performed within the same view field (Figure 1d) confirmed the expected C/Ti atomic concentration ratio of 2:3 for Ti_3_C_2_T*_x_* MXene besides the residual presence of Al (Table S2). The spectra also show O and F due to their presence as MXene surface terminations (T*_x_* = −OH, −O, −F, etc.). An example of SEM-EDX elemental mapping of Ti_3_C_2_T*_x_* MXene is shown in Figure S1.

**Figure 2:**
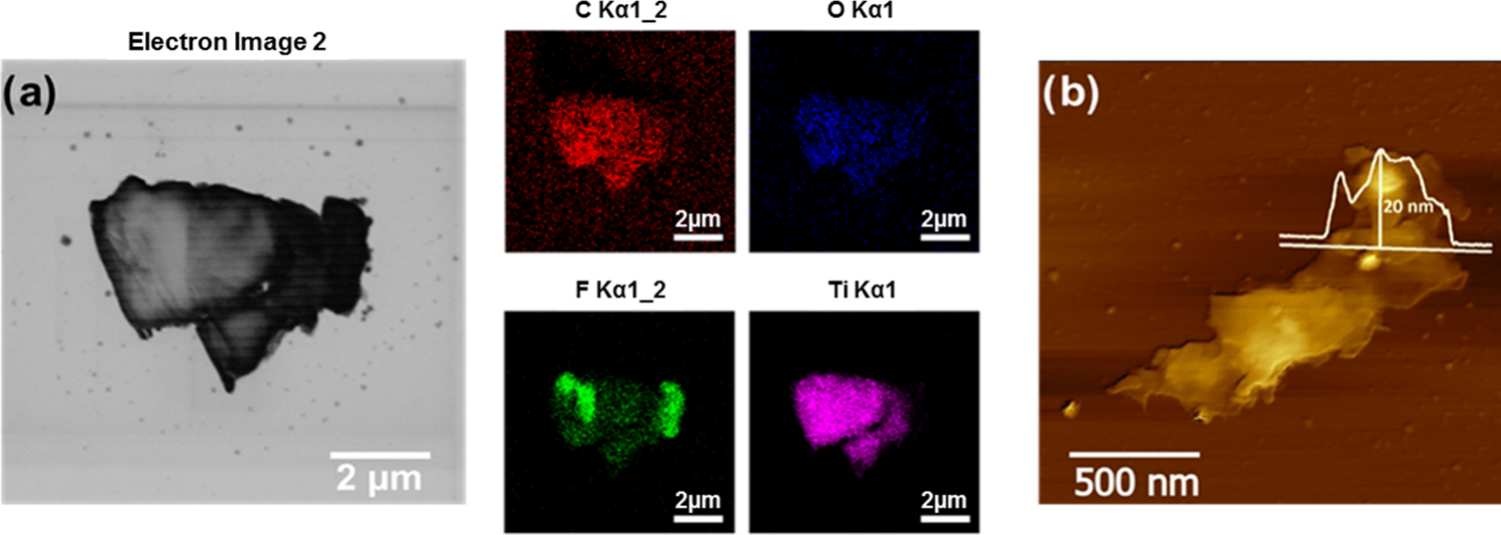
Characterization of Ti_3_C_2_T*_x_* MXene flakes: STEM image and respective mapping of elements; the scale bar represents 2 μm (a). AFM image with a height profile; the scale bar represents 500 nm (b).

Separated Ti_3_C_2_T*_x_* MXene flakes were obtained after sonication to form a stable suspension, as observed by STEM and shown in Figures 2a and S2, which typically exhibited micron size in the lateral scale. The elemental maps confirm the composition and distribution of Ti, C, and O, although there was a nonuniform structural feature of F in the Ti_3_C_2_T*_x_* MXene flake (Figure 2a). The selected elemental line profiles of Ti and C within the same region show a clear higher intensity of both elements within a length of 2.8 μm (Figure S3); positions with a higher Ti concentration match those with a higher C concentration. The AFM image (Figure 2b) shows the corresponding height profile of the Ti_3_C_2_T*_x_* MXene flake, up to 20 nm, with a lateral size of approximately 1.5 μm. The layer number can be estimated from the theoretical value for a single layer of Ti_3_C_2_T*_x_* (ca. 0.9 nm) and the measured flake thickness which points to ca. 22 layers. In practical terms, gaps occur between some of the layers, which can be seen in SEM images (Figures 1c and S1); thus, it is possible that the actual number of layers can be lower than the predicted.

### Surface Analysis

**Figure 3:**
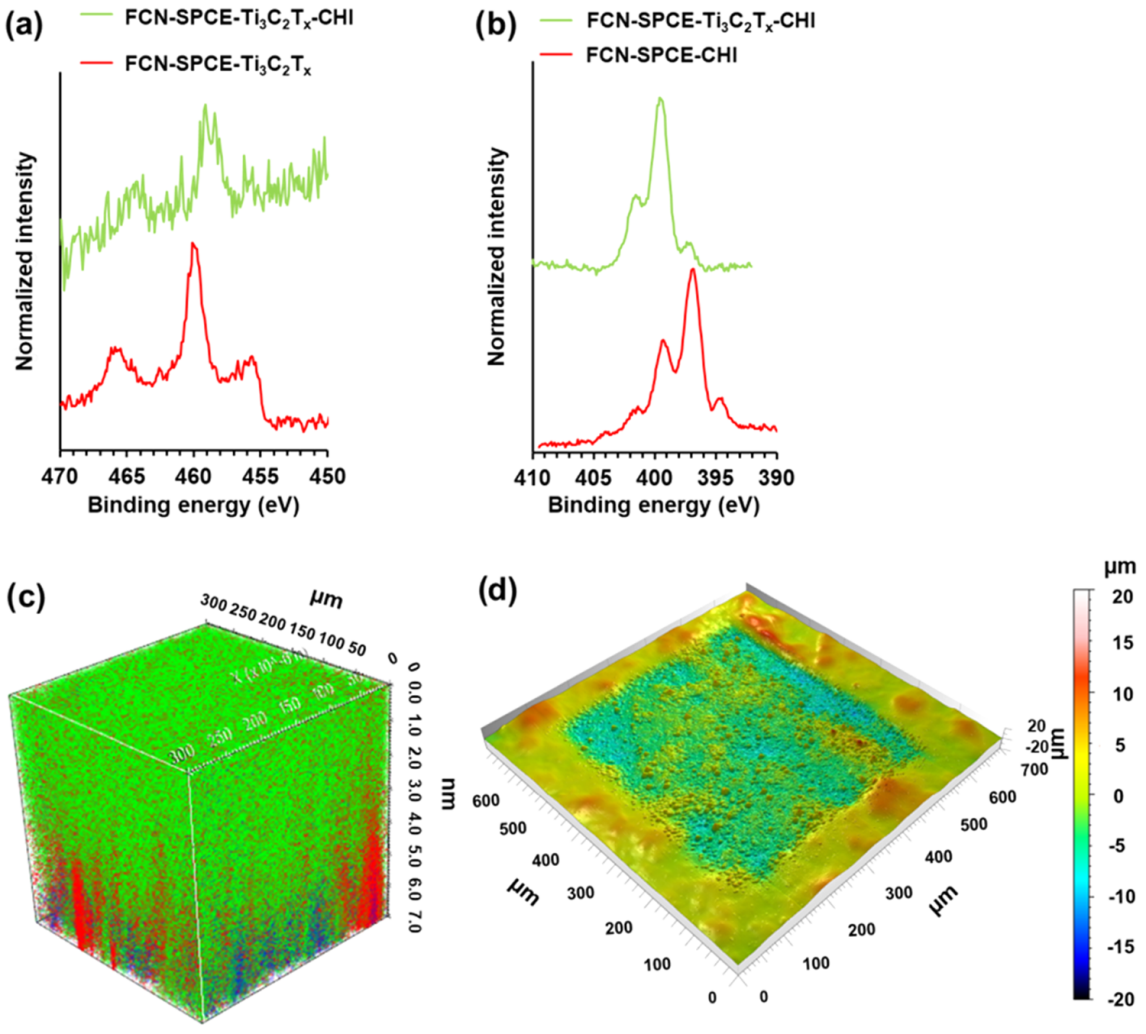
High-resolution Ti 2p (a) and N 1s (b) XPS spectra of FCN-SPCE-Ti_3_C_2_T*_x_*-CHI (green) and FCN-SPCE-Ti_3_C_2_T*_x_* (red) (a) and FCN-SPCE-Ti_3_C_2_T*_x_*-CHI (green) and FCN-SPCE-CHI (red) (b). 3D ToF-SIMS image of the FCN-SPCE coated with CHI (green), Ti_3_C_2_T*_x_* MXene (red), and ferrocyanide (blue) (c) and the sputter crater in the sensing membrane measured with a stylus profilometer (d).

Figure 3a shows high-resolution Ti 2p spectra for the ferrocyanide-modified screen-printed carbon electrode (FCN-SPCE) coated with Ti_3_C_2_T*_x_* (FCN-SPCE-Ti_3_C_2_T*_x_*) and the FCN-SPCE electrode coated with Ti_3_C_2_T*_x_* and chitosan (CHI), i.e., FCN-SPCE-Ti_3_C_2_T*_x_*-CHI. The main Ti 2p peak for FCN-SPCE-Ti_3_C_2_T*_x_* is located at 460.0 eV, whereas the Ti 2p spectrum for the electrode that was additionally coated with CHI, i.e., FCN-SPCE-Ti_3_C_2_T*_x_*-CHI, shifts to more negative binding energies, which suggest bonding of Ti-containing species and CHI.

A higher degree of noise is present in the Ti 2p spectrum for FCN-SPCE-Ti_3_C_2_T*_x_*-CHI as Ti_3_C_2_T*_x_* was underneath the CHI layer, and less signal was obtained for Ti 2p. Figure 3b shows N 1s spectra for the FCN-SPCE electrode coated with CHI (FCN-SPCE-CHI) and the FCN-SPCE electrode coated with Ti_3_C_2_T*_x_* and CHI (FCN-SPCE-Ti_3_C_2_T*_x_*-CHI). The main peak in the N 1s spectrum for FCN-SPCE-CHI is located at 397.0 eV, while this main peak for FCN-SPCE-Ti_3_C_2_T*_x_*-CHI is shifted to the more positive binding energy. Additional deconvolution of Ti 2p and N 1s spectra with peak assignments is given in the Supporting Information as Figure S5, along with the corresponding discussion. Both peak shifts in the Ti 2p and N 1s spectra indicate that an interaction between CHI and Ti_3_C_2_T*_x_* occurred, which can explain the electrochemical stabilization of MXene by CHI.

The remarkable stabilization effect was confirmed by the electrochemical characterization of Ti_3_C_2_T*_x_* MXene in the presence and absence of CHI, as shown in Figure S4. The study compares the square-wave voltammetric responses obtained with bare SPCE (black), CHI-modified SPCE (green), Ti_3_C_2_T*_x_*-modified SPCE (blue), and CHI-Ti_3_C_2_T*_x_*-modified SPCE (red) in 0.1 M KCl as the supporting electrolyte. The signal at ca +0.55 V can be attributed to the typical oxidation of Ti_3_C_2_T*_x_* MXene deposited on the SPCE.^13^ On the other hand, when the Ti_3_C_2_T*_x_* MXene was codeposited with CHI (red), no oxidation signal was observed at this potential, indicating a favorable stabilization effect of CHI. Clearly, the signal at ca +0.0 V belongs to CHI. In this study, a supporting SPCE was used instead of FCN-SPCE since the latter exhibits a redox activity in the potential window significant for both studied species, i.e., MXene and CHI.

The ToF-SIMS technique was employed to determine the spatial distribution of the species in the gas-sensing membrane. Signals for CH_4_N^+^ at *m*/*z* 30.03, C_2_H_4_NO^+^ at *m*/*z* 58.03, C_2_H_5_NO^+^ at *m*/*z* 59.04, C_3_H_6_NO^+^ at *m*/*z* 72.05, and C_6_H_10_NO_3_^+^ at *m*/*z* 144.09^54−57^ were used to determine the 3D distribution of chitosan. The spatial distribution of Ti_3_C_2_T*_x_* was characterized by the signal for Ti^+^ at *m*/*z* 47.95, and the position of ferrocyanide was characterized by the signal for Fe^+^ at *m*/*z* 55.94. The obtained depth profile was processed to obtain a 3D image, which is presented in Figure 3c. Chitosan covers the topmost position (green area with a thickness of ca 4 μm), followed by Ti_3_C_2_T*_x_* (red spots), which is deposited on the ferrocyanide-modified (blue spots) screen-printed carbon electrode. The ToF-SIMS 3D imaging, therefore, confirms the layered design of the sensor, whereas Figure 3d shows the topography of the sensing membrane and the depth of the sputter crater (ca 7 μm) measured by the stylus profilometer.

### Electroanalytical Performance

Since the initial experiments showed a positive effect of MXene upon the detection of gaseous H_2_O_2_, we investigated the cyclic voltammetric behavior of gas sensors that were prepared using different concentrations of MXene in the modification layer.

**Figure 4:**
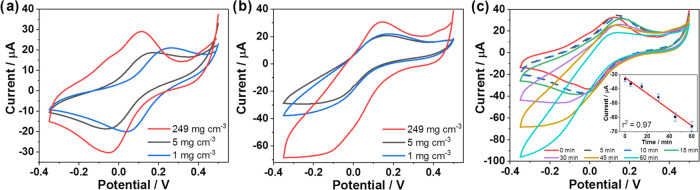
Cyclic voltammograms (CVs) recorded with the sensors prepared using different concentrations of MXene in the absence (a) and presence (b) of 10 mg m^–3^ gaseous H_2_O_2_ using a 20 min accumulation and a scan rate of 100 mV s^–1^. CVs recorded for successive increments of accumulation time in the range of 0–60 min together with a background response (red) for 5 mg m^–3^ gaseous H_2_O_2_ using a scan rate of 100 mV s^–1^ (c) (seven different sensors were used per each time point). All experiments were carried out using completely assembled sensors (FCN-SPCE-MXene-CHI).

Figure 4 shows the cyclic voltammetric responses of such gas sensors in the absence (a) and presence (b) of 10 mg m^–3^ gaseous H_2_O_2_. In the absence of H_2_O_2_, the sensor revealed two well-developed redox signals corresponding to the oxidation and reduction of Fe(CN)_6_^4–^/Fe(CN)_6_^3–^ redox couple between ca +0.00 and −0.20 V vs Ag quasi-reference electrode (Figure 4a). The most pronounced effect of MXene was observed at the highest concentration tested (249 mg cm^–3^) at the electrode surface, whereas the modification with 5 mg cm^–3^ MXene resulted only in shifted peaks of the Fe(CN)_6_^4–^/Fe(CN)_6_^3–^ redox couple toward more negative potentials for ca 100 mV. After only 20 min of exposure to gaseous H_2_O_2_, a substantial increase in the reduction signal was observed, as shown in Figure 4b.

In the presence of H_2_O_2_, (Fe(CN)_6_)^4–^ is chemically re-oxidized to (Fe(CN)_6_)^3–^, followed by immediate electrochemical re-reduction at the electrode surface. Consequently, the voltammogram displayed an increased reduction signal during the cathodic potential scan. It can be perceived that the highest examined concentration of MXene resulted in the highest cathodic response toward gaseous H_2_O_2_. On the other hand, it is also evident that the sensor containing the highest concentration of MXene exhibited a somewhat higher anodic signal, implying favorable electrocatalytic characteristics of MXene.

To gain further insight into the electroanalytical properties of the gas sensor, we investigated the effect of accumulation time on its voltammetric response; each measurement was performed with a newly fabricated sensor (Figure 4c). It was found that the current signal increased almost linearly with increasing accumulation time in the examined time range of 0–60 min. In this range, we did not observe any concentration saturation. On the contrary, apart from a linear increase of the signal (*r*^2^ = 0.97) at longer exposure times, excellent reproducibility was observed for each data point, as shown by the small error bars in Figure 4c. An even higher response could be achieved by further increasing the accumulation time; however, considerably longer accumulation times are associated with the inherent instability of gaseous H_2_O_2_.

With longer accumulation times and consequently with higher concentrations of gaseous H_2_O_2_ in the sensing membrane, the signal waveform gradually changes from a peak-shaped to a steady state-shaped voltammogram and then, with even longer accumulation times, to a situation with a well-visible deflection point. This process is also accompanied by a gradual shift of the reduction potential toward more negative values. This is due to the change in the diffusion profile with more accumulated H_2_O_2_ and electrogenerated product as a result of H_2_O_2_ reduction in the vicinity of the electrode surface, along with the inherently irreversible reduction reaction of H_2_O_2_.

**Figure 5:**
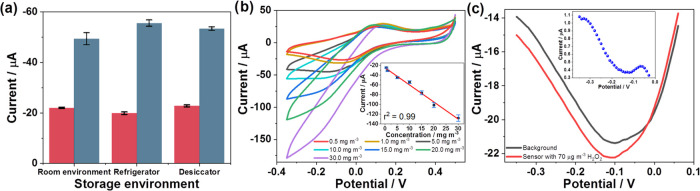
CV signal dependence on different storage conditions in the absence (red) and presence (blue) of 10 mg m^–3^ H_2_O_2_: refrigerator (ca 4 °C, 65% RH), laboratory (ca 26 °C, 59% RH), and desiccator (ca 26 °C, 10% RH) (a). CVs recorded for successive increments of gaseous H_2_O_2_ concentrations in the range of 0.5–30.0 mg m^–3^ using a 50 min accumulation and the corresponding calibration plot (b). CVs recorded for 70 μg m^–3^ gaseous H_2_O_2_ (red) together with background response (black) using a 60 min accumulation; a background subtracted voltammogram is shown in the inset (dotted blue) (c). Other conditions are as in Figure 4c.

In addition, the stability study of the sensor was conducted at various temperatures and humidity, as shown in Figure 5a. The sensors were stored overnight in different environments, i.e., in the laboratory, refrigerator, and desiccator; they were tested in triplicate. It can be observed that the background responses of the sensors stored in different ways are practically the same, i.e., within the range of standard deviation. Interestingly, storage in the desiccator did not affect the sensor response, i.e., the dry atmosphere did not compromise the electroanalytical performance of the gas sensor. A slightly lower response toward the gaseous analyte was observed when the sensor was stored under ambient conditions. This could be due to the combination of higher ambient humidity and temperature (at more than 5 °C), which could accelerate the hydrolytic damage degree of the chitosan and change its physicochemical properties.^58,59^

We followed the performance of the sensor in terms of its response to increasing concentrations of gaseous H_2_O_2_. In combination with a 50 min accumulation, the sensor exhibited a well-developed and satisfactory linear response in the examined concentration range of 0.5–30 mg m^–3^ with *r*^2^ of 0.99 (Figure 5b). The deviation from the higher linearity is due to the accumulation/dissolution/diffusion pattern of H_2_O_2_ in the viscous sensing membrane and the partial decomposition of relatively unstable H_2_O_2_.

The gas sensor was also successfully tested for the detection of lower H_2_O_2_ concentration; as depicted in Figure 5c, a well-readable voltammetric signal was obtained for 70 μg m^–3^ in conjunction with an accumulation time of only 60 min. The sensor unveiled a very low limit of detection (3σ criterion) of 4 μg m^–3^. Moreover, surprisingly, good sensor-to-sensor repeatability was achieved, with a relative standard deviation (RSD) of only 1.30% when measuring 70 μg m^–3^ gaseous H_2_O_2_.

### Selectivity Study

**Figure 6:**
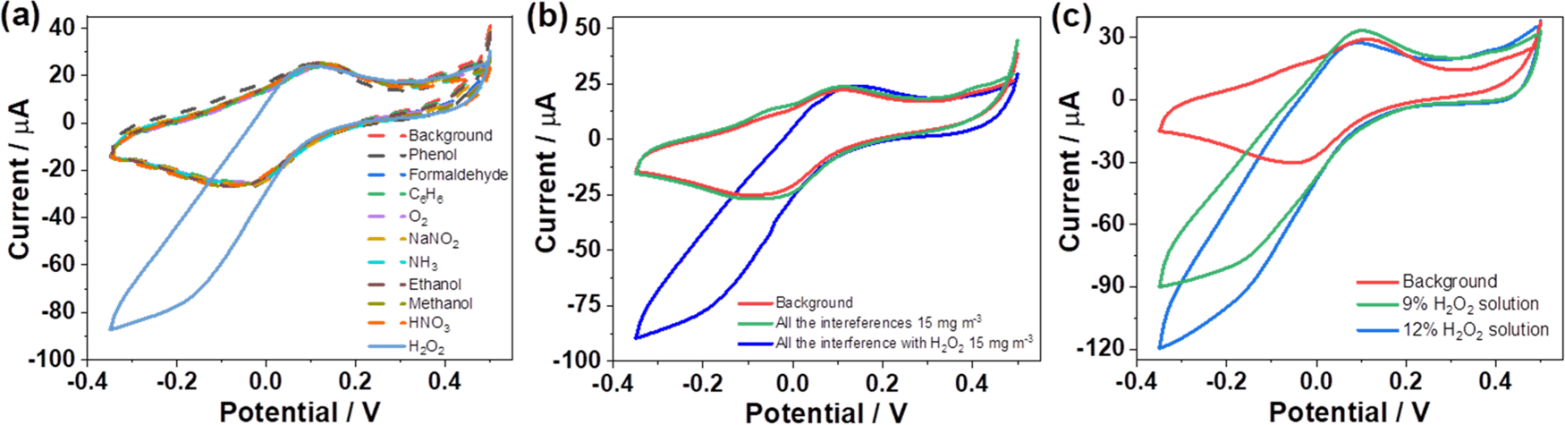
CVs recorded individually for nine potential gaseous interferants of 15 mg m^–3^ each (a) and in the presence of all nine interferants together (of 15 mg m^–3^ each) (b), along with the addition of 15 mg m^–3^ H_2_O_2_ and a background response using a 20 min accumulation. CVs recorded for a background (red) and when the gas sensor was left for 20 min in a room, where hair bleaching was carried out using 9% (green) and 12% (blue) H_2_O_2_ solution (c). Other conditions are as in Figure 4c.

The operation of the gas sensor was investigated in the presence of selected potential gaseous compounds expected in the real environment. The study was performed by measuring the potential interferants individually at a concentration of 15 mg m^–3^ each, followed by the addition of 15 mg m^–3^ H_2_O_2_, as shown in Figure 6a.

In the absence of gaseous H_2_O_2_, no analytical signal was detected for gaseous methanol, ethanol, formaldehyde, phenol, ammonia, benzene, oxygen, nitric acid, and sodium nitrite in the applied potential range of +0.50 to −0.35 V. In addition, the sensor was exposed to a mixture of all of these gaseous compounds at a concentration of 15 mg m^–3^ each. After the addition of 15 mg m^–3^ H_2_O_2_, a well-defined reduction signal of H_2_O_2_ was formed with no visible effects of the nine gaseous compounds present, confirming the excellent selectivity of the gas sensor (Figure 6b).

### Real Sample Detection

Finally, the performance of the gas sensor was tested in a real environment during the bleaching treatment of human hair. The experiment was carried out in a fume hood by applying 10.0 mL of a 9% H_2_O_2_ solution to the wig; each of the three sensors was exposed for 20 min. After washing and drying the wig, the same procedure was repeated with 10.0 mL of a 12% H_2_O_2_ solution (Figure 6c). In both cases, the background was measured in the absence of gaseous H_2_O_2_. The sensor exhibited distinct signals for both concentrations of gaseous H_2_O_2_; after three successive measurements, an average determined concentration of ca 18 mg m^–3^ for 9% H_2_O_2_ solution and ca 24 mg m^–3^ for 12% H_2_O_2_ solution was obtained.

## Conclusions

In this work, we have demonstrated a synergistic effect of the advantageous physicochemical properties of exfoliated Ti_3_C_2_T*_x_* MXene and the stabilizing characteristics of chitosan, together with the redox activity of the ferrocyanide-modified screen-printed carbon electrode for ultrasensitive point-of-interest detection of gaseous H_2_O_2_. The poly(acrylic acid)-based membrane served simultaneously as an electrolyte and a suitable accumulation medium for the gaseous analyte. The sensor showed excellent interference-free operation in the cathodic potential range along with good sensor-to-sensor reproducibility, favorable stability, and a very low detection limit of only 4 μg m^–3^ (ppbv). The applicability of the Ti_3_C_2_T*_x_* MXene-based gas sensor was successfully demonstrated by measuring H_2_O_2_ during the treatment of real human hair with a bleaching agent.

## Supporting Information Available

Synthesis route to prepare Ti_3_C_2_T*_x_* MXene from the precursor Ti_3_AlC_2_ MAX phase (Scheme S1); SEM micrograph of Ti_3_C_2_T*_x_* MXene and mapping of elements (Figure S1); bright-mode STEM image of Ti_3_C_2_T*_x_* MXene flakes (Figure S2); mapping of elements of Ti_3_C_2_T*_x_* MXene flakes and relative intensity profiles (Figure S3); Raman shift positions for Raman-active modes of Ti_3_C_2_T*_x_* (Table S1); EDXS element quantification of Ti_3_C_2_T*_x_* MXene (Table S2); square-wave voltammograms of MXene stabilization study (Figure S4); and deconvolution of the high-resolution Ti 2p XPS spectra and N 1s XPS spectra for sensing layers (Figure S5) (PDF)
