# Correction to “Study of Chitosan-Stabilized Ti_3_C_2_T_*x*_ MXene for Ultrasensitive and Interference-Free Detection of Gaseous H_2_O_2_”

**DOI:** 10.1021/acsami.3c10281

**Published:** 2023-08-03

**Authors:** Jelena Isailović, Ana Oberlintner, Uroš Novak, Matjaž Finšgar, Filipa M. Oliveira, Jan Paštika, Zdeněk Sofer, Nikola Tasić, Rui Gusmão, Samo B. Hočevar

In the original version of the article (https://pubs.acs.org/doi/10.1021/acsami.3c05314), on p. 31645, Figure 1a contains an incorrectly assigned XRD peak (008). Namely, the sharp diffraction pattern above 30° originates from TiC and nonetched Ti_3_AlC_2_. In the revised Figure 1 shown here, the mark (008) in Figure 1a is deleted, and accordingly, the discussion on p. 31645 is changed to “The XRD pattern of Ti_3_C_2_T_*x*_ MXene is shown in Figure 1a, and its successful synthesis is confirmed by the presence of characteristic diffraction peaks at 8.7°, 18.2°, and 27.6° which are related to the (002), (004), and (006) planes of this MXene, respectively. The sharp diffraction pattern above 30° originates from TiC and nonetched Ti_3_AlC_2_.”

**Figure 1:**
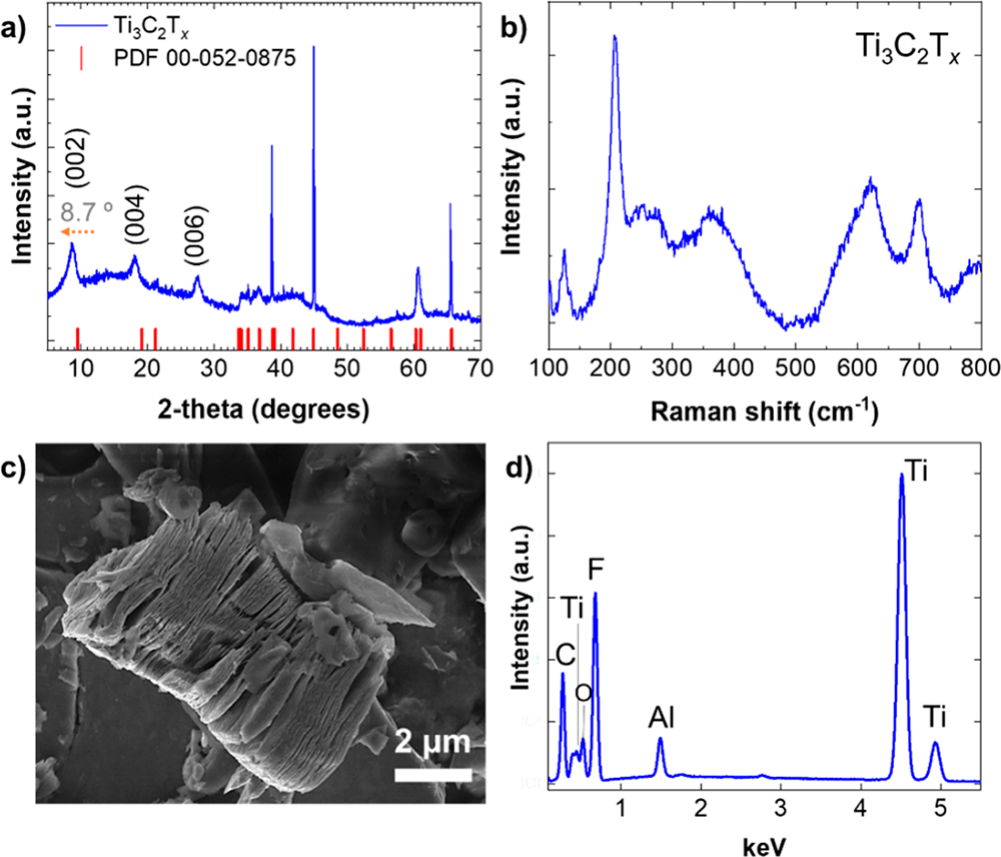
Structural and morphological characterization of Ti_3_C_2_T_*x*_ MXene by XRD (a), Raman spectroscopy (b), SEM (c), and EDXS (d).

In the original version of the article, on p. 31649, in the Acknowledgments, the sentence: “Z.S. was supported by Czech Science Foundation (GAčR No. 20-16124J)” is replaced by “Z.S. was supported by ERC-CZ program (project LL2101) from Ministry of Education Youth and Sports (MEYS). F.M.O. would like to acknowledge the funding from MEYS under the CHEMFELLS V Project No. CZ.02.01.01/00/22_010/0003004.”

In the original Supporting Information (SI), Figure S3a and b, the scale bars should be 2 μm, not 2.5 μm. A revised SI file is included here with a revised Figure S3 and the following text added at the end of the Figure S3 caption: “Scale bars represent 2 μm.”

Also, in the original SI file, p. 6, please note that all mentions of Figure 5 should be read Figure S5. This has been fixed in the attached SI file.

With these changes, the conclusions of the work have not been affected.

## Supporting Information Available

Same description as in the original text (PDF)

