# Supplementary material for: Study of Chitosan-Stabilized Ti3C2Tx MXene for Ultrasensitive and Interference-Free Detection of Gaseous H2O2
Source: ACS Appl Mater Interfaces. 2023 Jun 23;15(26):31643–51. doi: 10.1021/acsami.3c05314 (PMC10326801; doi:10.1021/acsami.3c05314)
Supplement: Supplementary file 1 — am3c05314_si_001.pdf [file am3c05314_si_001.pdf]

Supporting Information

# Study of Chitosan-Stabilized $Ti_3C_2T_x$ MXene for Ultrasensitive and Interference-Free Detection of Gaseous $H_2O_2$

*Jelena Isailović,[a,c] Ana Oberlintner,[c,d] Uroš Novak,[d] Matjaž Finšgar,[e] Filipa M. Oliveira,[b] Jan Paštika,[b] Zdeněk Sofer,[b] Nikola Tasić,[a] Rui Gusmão,[b,*] Samo B. Hočevar [a,*]*

[a] Department of Analytical Chemistry, National Institute of Chemistry, Hajdrihova 19, 1000 Ljubljana, Slovenia

[b] Department of Inorganic Chemistry, University of Chemistry and Technology Prague, Technická 5, 166 28 Prague, Czech Republic

[c] International Postgraduate School Jožef Štefan, Jamova cesta 39, 1000 Ljubljana, Slovenia

[d] Department of Catalysis and Chemical Reaction Engineering, National Institute of Chemistry, Hajdrihova 19, 1000 Ljubljana, Slovenia

[e] Faculty of Chemistry and Chemical Engineering, University of Maribor, Smetanova ulica 17, 2000 Maribor, Slovenia

*Samo. B. Hočevar (e-mail: samo.hocevar@ki.si)

*Rui Gusmão (e-mail: rui.gusmao@vscht.cz)

**KEYWORDS:** $Ti_3C_2T_x$ MXene, Chitosan, Hydrogen peroxide, Gas sensor, Cyclic Voltammetry.

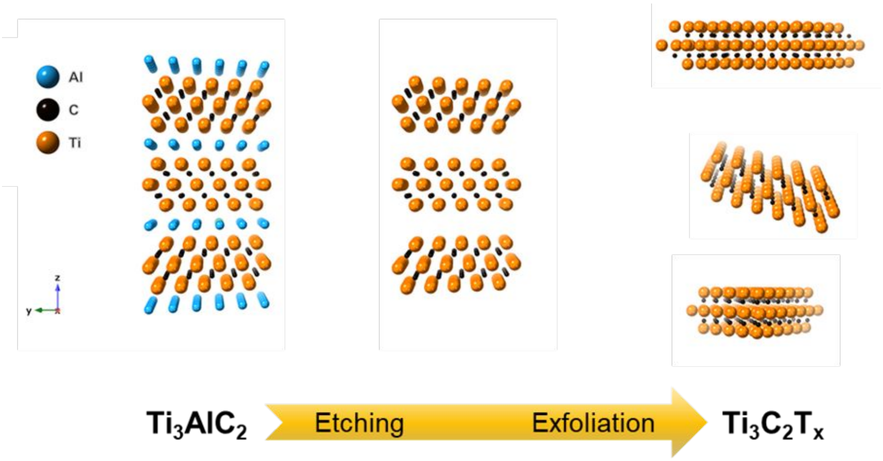


**Scheme S1.** Representation of the synthesis route to prepare $Ti_3C_2T_x$ MXene from the precursor $Ti_3AlC_2$ MAX phase.

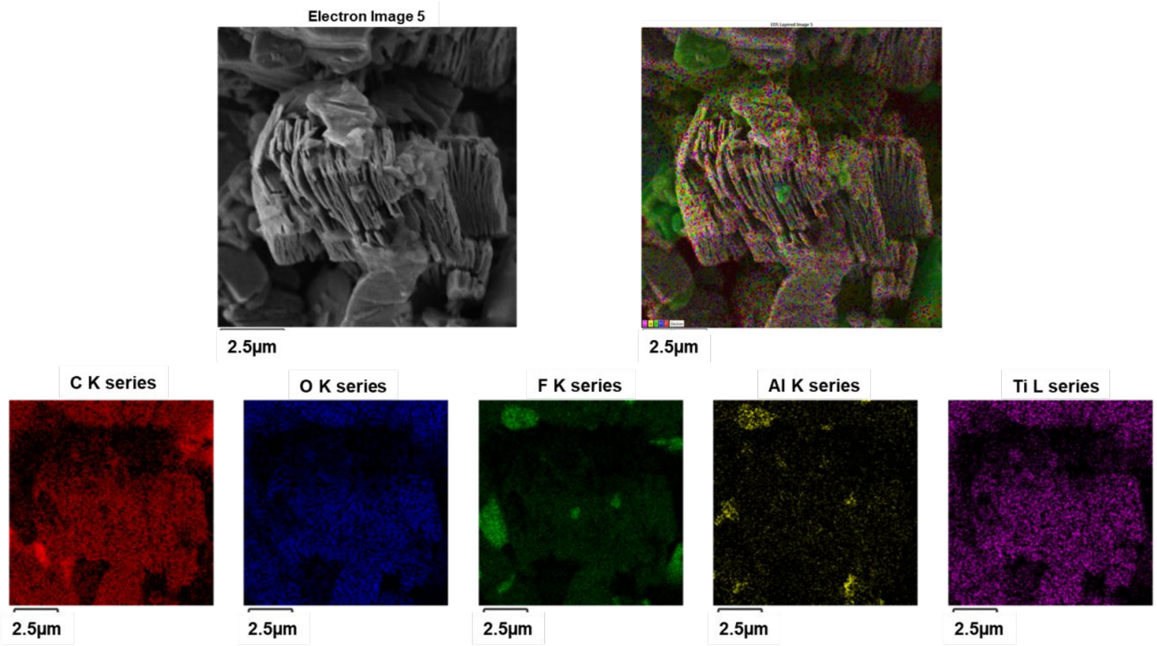


**Figure S1.** SEM micrograph of $Ti_3C_2T_x$ MXene (top left) with the overlay colored mapping of elements (top right). Individual mapping of elements for C, O, F, Al, and Ti (bellow). The scale bar represents 2.5 µm.

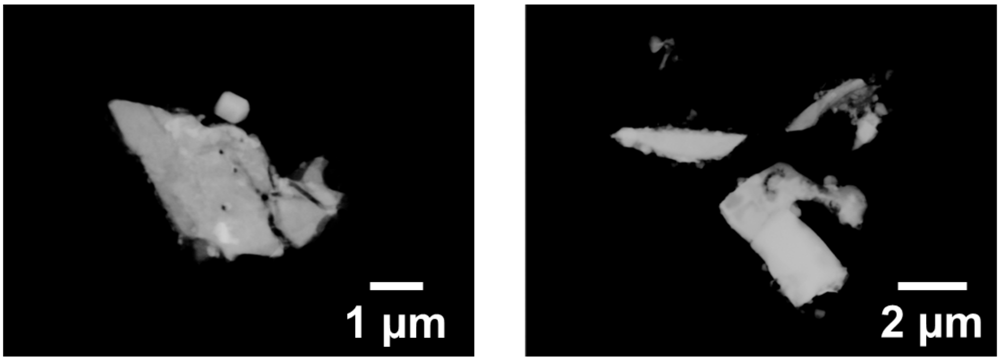


**Figure S2.** Bright mode STEM image of $Ti_3C_2T_x$ MXene flakes.

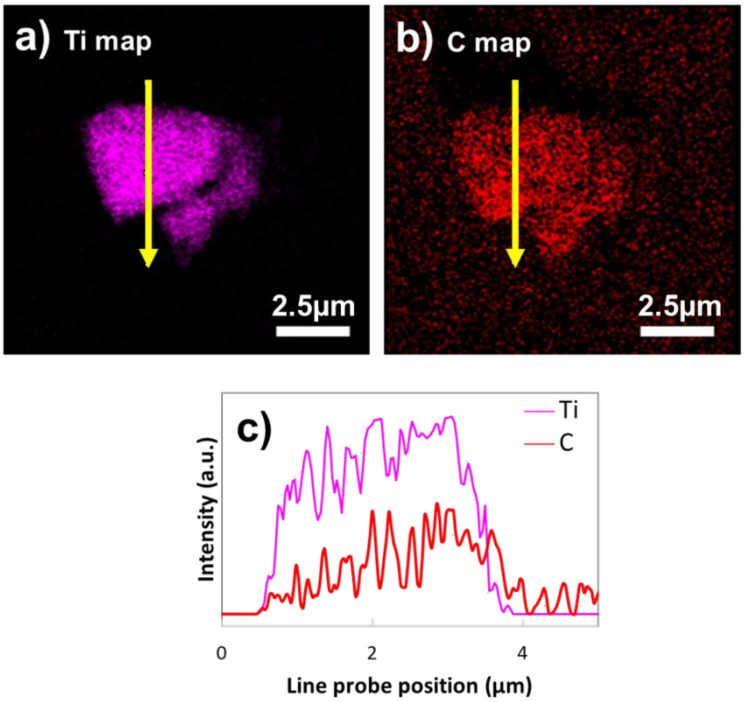


**Figure S3.** Mapping of elements of $Ti_3C_2T_x$ MXene flakes: Ti (a), C (b). The yellow arrow denotes the EDXS elemental line probe for Ti and C elements for which the relative intensity profiles were obtained (c).

**Table S1.** Raman shift positions for Raman-active modes of $Ti_3C_2T_x$. All the values are given in $cm^{-1}$.

| **Material** | **Resonant peak** | $A_{1g}$ (Ti, C, O) | $E_g$ (C) | $A_{1g}$ (C) | **Ref.** |
|---|---|---|---|---|---|
| $Ti_3C_2T_x$ | 126.1 | 206.5 | 621.3 | 700.3 | **This work** |
| $Ti_3C_2T_x$ (HF-HCl Multilayer) | 122.2 | 202.8 | ~ 620 | 721.8 | 1 |
| $Ti_3C_2T_x$ (DFT calculations) | - | 228 | 621 | - | 2 |

[52] Sarycheva, A.; Gogotsi, Y. Raman Spectroscopy Analysis of the Structure and Surface Chemistry of $Ti_3C_2T_x$ MXene. *Chem. Mater.* 2020, *32* (8), 3480–3488.
[53] Hu, T.; Wang, J.; Zhang, H.; Li, Z.; Hu, M.; Wang, X. Vibrational Properties of $Ti_3C_2$ and $Ti_3C_2T_2$ (T = O, F, OH) Monosheets by First-Principles Calculations: A Comparative Study. *Phys. Chem. Chem. Phys.* 2015, *17* (15), 9997–10003.

**Table S2.** EDXS element quantification of $Ti_3C_2T_x$ MXene.

| at.% | C | O | F | Al | Ti |
|---|---|---|---|---|---|
| **Average** | 19.6 | 19.2 | 31.2 | 1.7 | 28.2 |
| **Standard deviation** | 2.3 | 1.9 | 2.4 | 0.2 | 3.1 |

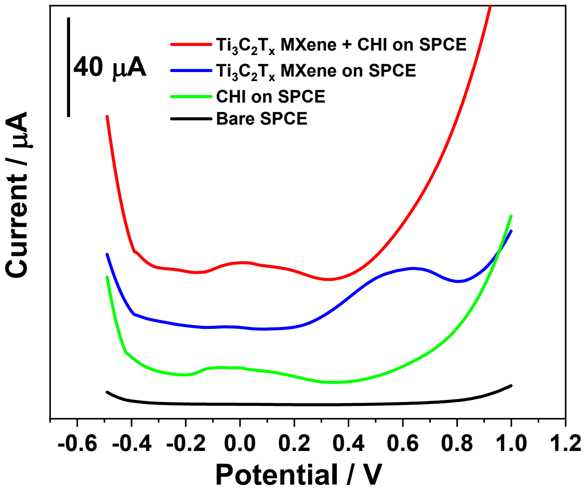


**Figure S4.** Square-wave voltammograms recorded in 0.1 M KCl using bare SPCE (black), CHI-modified SPCE (green), $Ti_3C_2T_x$ MXene-modified SPCE (blue), and $Ti_3C_2T_x$ MXene + CHI-modified SPCE (red), using an amplitude of 100 mV, a frequency of 20 Hz and a step potential of 10 mV.

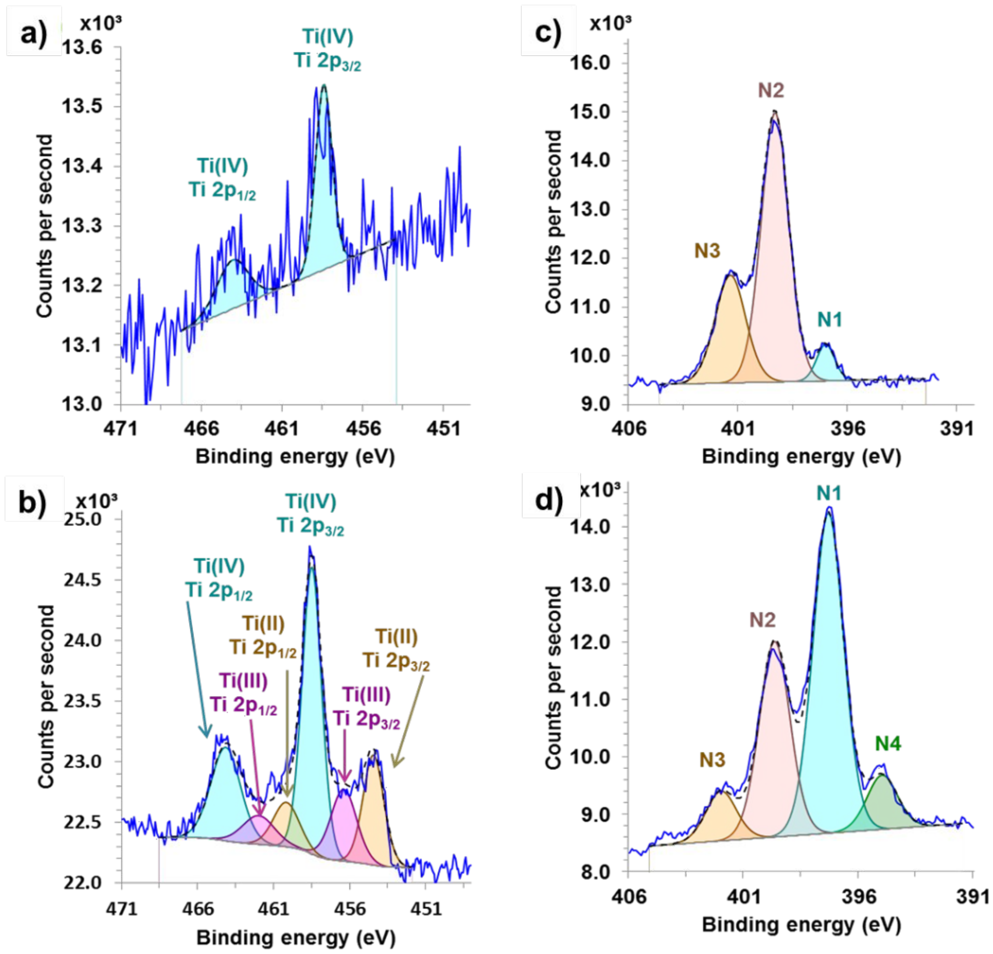


**Figure S5.** Deconvolution of the high-resolution Ti 2p XPS spectra for FCN-SPCE-$Ti_3C_2T_x$-CHI (a) and FCN-SPCE-$Ti_3C_2T_x$ (b), and deconvolution of the high-resolution N 1s XPS spectra for FCN-SPCE-$Ti_3C_2T_x$-CHI (c) and FCN-SPCE-CHI (d).

Figure 5 shows deconvoluted Ti 2p spectra for FCN-SPCE-$Ti_3C_2T_x$-CHI (Figure 5a) and FCN-SPCE-$Ti_3C_2T_x$ (Figure 5b). A higher degree of noise is present in Figure 5a due to the coverage of the surface (also Ti-containing species) with CHI. The positions of the Ti $2p_{3/2}$ and Ti $2p_{1/2}$ peaks correspond to Ti(IV) oxidation state. In the case of FCN-SPCE-$Ti_3C_2T_x$, i.e., the sample that was not covered with CHI, the Ti 2p spectrum is different and can be fitted with three oxidation states, i.e., Ti(IV), Ti(III), and Ti(II) (Figure 5b). As this sample was not covered with CHI, the XPS excitation signal also reached regions with Ti(II) and Ti(III) oxidation states of the MXene. The peaks for the Ti(II) and Ti(III) oxidation states were less intense compared to the peak for Ti(IV), implying lower surface atomic concentrations of these species compared to Ti(IV)-containing species. In the case of FCN-SPCE-$Ti_3C_2T_x$-CHI, Ti(II)- and Ti(III)-containing species were deeper in the subsurface

region compared to Ti(IV)-containing species, and the XPS excitation signal did not reach them (hence the peaks for these species are missing in Figure 5a).

Figures 5c and 5d show deconvoluted N 1s spectra for FCN-SPCE-$Ti_3C_2T_x$-CHI and FCN-SPCE-CHI, respectively. Deconvoluted peaks designated as N3 and N2 correspond to $NH_3^+$ and $NH_2$ in the CHI molecule. The deconvoluted peak N1 might originate from nonbounded hexacyanoferrate, and the deconvoluted peak N4 to hexacyanoferrate bonded to SPCE. The N4 peak in the case of FCN-SPCE-$Ti_3C_2T_x$-CHI (Figure 5c) was not developed, which is most likely due to the too-thick surface layer (as an additional layer of $Ti_3C_2T_x$ was present), and the XPS excitation signal did not reach the SPCE, where the connection of hexacyanoferrate with SPCE occurred. The same is most likely the reason for a less intense N1 peak in the case of FCN-SPCE-$Ti_3C_2T_x$-CHI (Figure 5c) compared to FCN-SPCE-CHI (Figure 5d), where more signal for nonbounded hexacyanoferrate was obtained for the latter as the surface layer was thinner (as MXene was not present).
